# A comparative analysis of hepatic pathological phenotypes in C57BL/6J and C57BL/6N mouse strains in non-alcoholic steatohepatitis models

**DOI:** 10.1038/s41598-018-36862-7

**Published:** 2019-01-18

**Authors:** Eri Kawashita, Keiichi Ishihara, Madoka Nomoto, Mika Taniguchi, Satoshi Akiba

**Affiliations:** Department of Pathological Biochemistry, Kyoto Pharmaceutical University, 5 Misasaginakauchi-cho, Yamashina-ku, Kyoto, 607-8414 Japan

## Abstract

C57BL/6J (BL6J) and C57BL/6N (BL6N) inbred substrains are most widely used to understand the pathological roles of target molecules in a variety of diseases, including non-alcoholic steatohepatitis (NASH), based on transgenic mouse technologies. There are notable differences in the metabolic phenotypes, including glucose tolerance, between the BL6J and BL6N substrains, but the phenotypic differences in NASH are still unknown. We performed a comparative analysis of the two mouse substrains to identify the pathological phenotypic differences in NASH models. In the CCl_4_-induced NASH model, the BL6J mice exhibited a more severe degree of oxidative stress and fibrosis in the liver than the BL6N mice. In contrast, in the high-fat diet-induced NASH model, more accumulation of hepatic triglycerides but less weight gain and liver injury were noted in the BL6J mice than in the BL6N mice. Our findings strongly suggest caution be exercised with the use of unmatched mixed genetic background C57BL6 mice for studies related to NASH, especially when generating conditional knockout C57BL6 mice.

## Introduction

Non-alcoholic fatty liver disease (NAFLD) is the most common chronic liver disease covering a spectrum of histopathological changes in the liver ranging from simple steatosis to non-alcoholic steatohepatitis (NASH) that may progress to hepatic fibrosis, cirrhosis, or hepatocellular carcinoma^1–3^. In the discovery of molecular mechanisms and new drugs for NAFLD/NASH, a number of mouse models have been used: gene-deleted (e.g. Alms1^−/−^ or Mc4r^−/−^), high-fat and -carbohydrate diet-fed, nutrient deficient diet-fed, and CCl_4_ chronically administered mouse models^4^. Genetic and lifestyle factors can lead to obesity, insulin resistance, and disorders of lipid metabolism, resulting in the accumulation of free fatty acids in the liver and, as a consequence, mitochondrial dysfunction with oxidative stress, endoplasmic reticulum (ER) stress, hepatocyte cell death, and the production of inflammatory chemokines and cytokines, such as monocyte chemotactic protein-1 (MCP-1) and tumor necrosis factor (TNF-α). Thus, the multiple-hit hypothesis is the most widely accepted explanation of the mechanisms underlying the progression of NASH^5,6^.

Genetically modifying systems, including transgenic and knockout technologies, have been utilized to understand the pathological roles of target molecules in a variety of diseases, including NAFLD/NASH^7^. One of the most widely used mouse strains is the C57BL/6, with more than 20 inbred substrains derived from C57BL/6J (BL6J) with “J” for Jackson and C57BL/6N (BL6N) with “N” for NIH. It has become clear that there are multiple genetic differences between the BL6J and BL6N substrains. A whole-genome sequence comparison between the substrains identified 34 coding single-nucleotide polymorphisms (SNPs), leading to amino acid substitutions in the encoded protein, 2 coding small insertions or deletions (indels), 146 noncoding SNPs, 54 noncoding small indels, and 43 structural variants including the nicotinamide nucleotide transhydrogenase (Nnt) mutation^8,9^. The most widely known difference is the spontaneous deletion of exon 7–11 in the *Nnt* gene, resulting in a complete absence of NNT, in the BL6J substrains, but not in the BL6N substrains^10^. Recently, Mekada *et al*. showed that even the BL6N-derived substrains (C57BL/6NJ, C57BL/6Ntac and C57BL/6NCrSlc), have identified SNPs^11^.

These small genetic differences between the BL6J and BL6N substrains lead to notable differences in the metabolic phonotypes, including differences in the glucose tolerance, insulin secretion, weight regulation, energy expenditure, and O_2_ consumption^9^. These findings suggest that caution be exercised regarding the use of mice with mixed BL6J and BL6N genetic backgrounds in research on metabolic syndromes, such as diabetes. However, the phenotypic differences between the BL6J and BL6N strains in NASH models remain unclear.

In the present study, we aimed to elucidate the pathological differences between these two substrains.

## Results

### Greater CCl_4_-induced hepatic oxidative stress in BL6J mice than in BL6N mice

**Figure 1:**
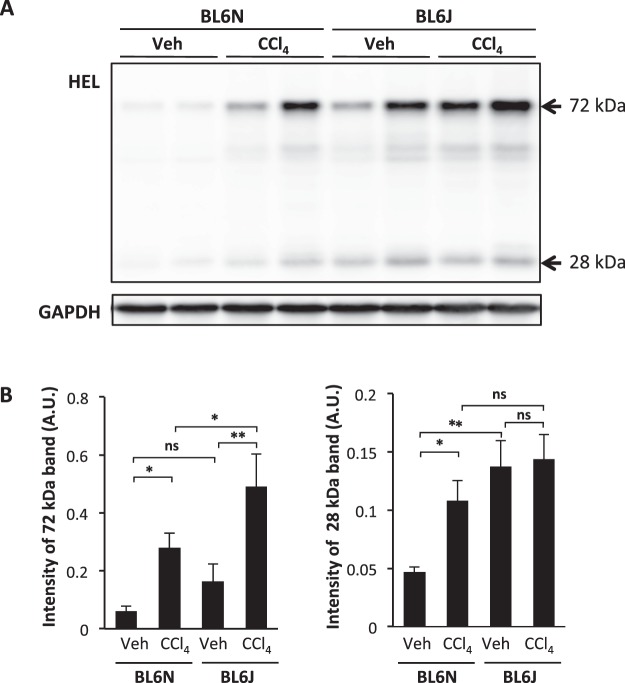
CCl_4_-induced oxidative stress in BL6J and BL6N mice. BL6J and BL6N mice were intraperitoneally administered CCl_4_ in corn oil at 0.31 mL/kg (2 times/week) for 6 weeks. (**A**) Levels of 13-HPODE-adducted protein relative to those of GAPDH in the livers of BL6J and BL6N mice were determined by Western blotting. The cropped blots were displayed in (**A**) and the full-length blots were presented in Supplementary Fig. 2. (**B**) The relative intensity was measured using the NIH ImageJ software program and normalized to that of GAPDH. The bar graphs represent the means ± SE (arbitrary units: A.U., n = 8–10/group). Significance was evaluated using an ANOVA with the LSD post-hoc test. *P < 0.05, **P < 0.01, ns: non-significant.

Trichloromethyl radical (.CCl_3_) metabolized from CCl_4_ by CYP2E1 in hepatocytes evokes lipid peroxidation and hepatotoxicity^12,13^. We confirmed that there was no marked difference in the mRNA expression of Cyp2e1 between the BL6J and BL6N mice (Supplementary Fig. 1). The degree of lipid peroxidation was assessed by detecting the liver levels of 13-hydroperoxyoctadecanoic acid (13-HPODE)-modified proteins, which reacts specifically with an anti-HEL antibody (Fig. 1 and Supplementary Fig. 2). The intensity of a band of approximately 72 kDa was increased by chronic CCl_4_ administration, and the increased level was significantly higher in the BL6J mice than in the BL6N mice, indicating that more severe oxidative stress was induced by CCl_4_ administration in the BL6J mice than in the BL6N mice. In addition, the level of the 13-HPODE-modified protein of 28 kDa was markedly higher in the BL6J mice than in the BL6N mice in vehicle-injected groups, suggesting that the BL6J mice have greater oxidative stress than BL6N mice even at the basal level.

### Comparable CCl_4_-induced hepatotoxicity and liver inflammation between the BL6 substrains

**Figure 2:**
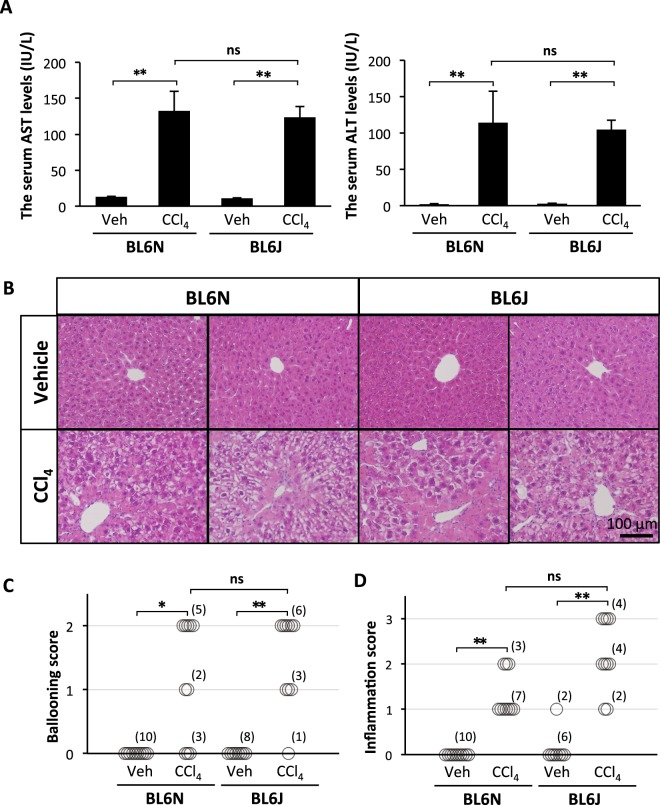
CCl_4_-induced liver injury and hepatic inflammation in BL6J and BL6N mice. (**A**) The serum levels of AST and ALT were determined using enzymatic assays. The bar graphs represent the means ± SE (n = 8–10/group). Significance was evaluated using an ANOVA with the LSD post-hoc test. **P < 0.01. (**B**) Paraffin-embedded liver sections were stained with HE. (**C** and **D**) Hepatocyte ballooning and hepatic inflammation scores were evaluated as detailed in the Materials and Methods. Significance was evaluated using the Kruskal-Wallis test. *P < 0.05, **P < 0.01.

The degree of CCl_4_-induced hepatotoxicity in the BL6J and BL6N mice was compared by measuring the serum levels of AST and ALT, markers of liver injury. As shown in Fig. 2A, the serum levels of AST and ALT were significantly increased by chronic CCl_4_ administration, but there were no marked differences in the levels of AST or ALT between the BL6J and BL6N mice. A histological analysis of liver sections showed that CCl_4_-induced hepatic damage and inflammation were observed in the perivascular area at approximately the same level in the both strains (Fig. 2B). The liver specimens were scored for the severity of hepatocyte ballooning and leukocyte infiltration (Fig. 2C,D). The degrees of the CCl_4_-induced ballooning and inflammation were not markedly different between the BL6J and BL6N mice, although the severity of the inflammation was likely higher in the BL6J mice than in the BL6N mice. These results suggest that there is little difference in CCl_4_-induced hepatotoxicity and liver inflammation between the BL6J and BL6N mice.

### Severe CCl_4_-induced hepatic fibrosis in BL6J mice compared with BL6N mice

**Figure 3:**
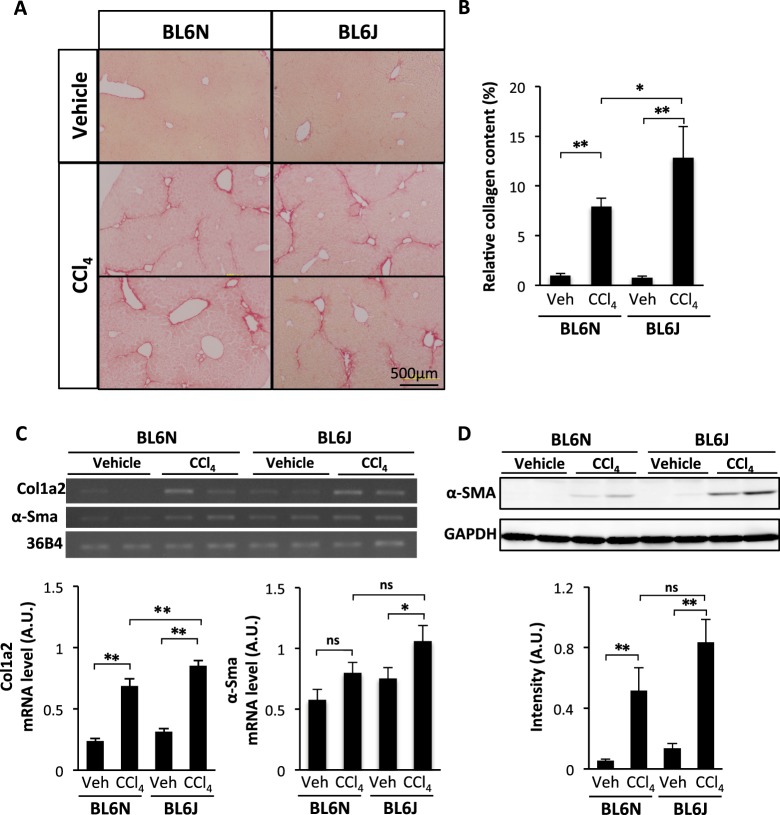
CCl_4_-induced hepatic fibrosis in BL6J and BL6N mice. (**A**) Liver sections were stained with picrosirius red. (**B**) The relative red-stained area was measured as the relative collagen content using the NIH ImageJ software program. (**C**) The mRNA expression of Col1a2 and α-Sma in the liver was determined by RT-PCR. The cropped gels were displayed in (**C**) and the full-length gels were presented in Supplementary Fig. 3. (**D**) The protein expression of α-SMA in the liver was determined by a Western blot analysis. The cropped blots were displayed in (**D**), and the full-length gels were presented in Supplementary Fig. 4. The band intensity was measured using the NIH ImageJ software program and normalized to that of 36B4 or GAPDH. The bar graphs represent the means ± SE (arbitrary units: A.U., n = 8–10/group). Significance was evaluated using an ANOVA with the LSD post-hoc test. *P < 0.05, **P < 0.01.

Hepatic fibrosis in the BL6J and BL6N mice was assessed by picrosirius red staining. Marked collagen deposition was observed surrounding the portal vein in the both BL6J and BL6N mice injected with CCl_4_ (Fig. 3A,B). The BL6J mice had a significantly higher level of collagen deposition than the BL6N mice. In addition, RT-PCR indicated that the collagen1a2 (Col1a2) mRNA level in the livers of the BL6J mice was also higher than that of the BL6N mice (Fig. 3C and Supplementary Fig. 3). Chronic CCl_4_ administration leads to the transformation of hepatic stellate cells (HSC) to myofibroblast-like cells expressing α-SMA, and the activated HSC produce collagen^14,15^. RT-PCR and Western blot analyses showed that the induction of α-SMA expression in the liver by CCl_4_ administration tended to be higher in the BL6J mice than in the BL6N mice (Fig. 3C,D, and Supplementary Figs 3 and 4).

### Less weight gain and liver injury, but more hepatic triglyceride accumulation in BL6J mice than in BL6N mice in HFD-induced NASH model

**Figure 4:**
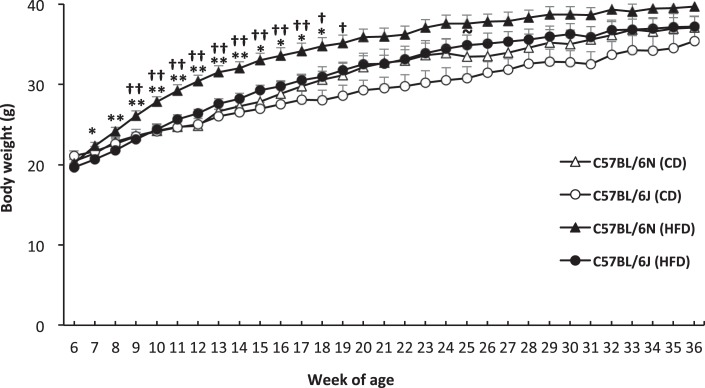
Body weight variation in BL6J and BL6N mice on an HFD. Mice were fed a control diet (CD; 4.3% fat) or a high-fat diet (HFD; 19.9% fat and 2.0% cholesterol) for 30 weeks, and the body weights were measured every week. The data represent the means ± SE (n = 7/group). Significance was evaluated using an ANOVA with the LSD post-hoc test. ^†^p < 0.05, ^††^p < 0.01 versus CD-fed BL6N; ^~^p < 0.05 versus CD-fed BL6J; *P < 0.05, **P < 0.01 versus HFD-fed BL6J.

To next elucidate the strain-related differences in HFD-induced weight gain and hepatology, BL6J and BL6N mice were randomly divided into two groups, and then fed a CD or HFD for 30 weeks. There was no marked difference in the amount of food consumed between the BL6J and BL6N mice; 3.23 ± 0.23 g in the CD-fed BL6N mice, 3.03 ± 0.09 g in the HFD-fed BL6N mice, 3.13 ± 0.09 g in the CD-fed BL6J mice, 3.00 ± 0.26 g in the HFD-fed BL6J mice. In the BL6N mice fed an HFD, a dramatic increase in the body weight was observed compared with the BL6J mice on an HFD, especially over the first nine weeks (Fig. 4), suggesting that the BL6J mice are more tolerant to HFD-induced weight gain than BL6N mice, especially in the initial period. In contrast to the significant findings for the body weight, the left lateral liver lobe weight/body weight ratios were 0.016 ± 0.0052 g in the CD-fed BL6N mice, 0.0337 ± 0.0047 g in the HFD-fed BL6N mice (p < 0.01 versus the CD-fed BL6N mice by an ANOVA with the LSD post-hoc test), 0.0168 ± 0.0057 g in the CD-fed BL6J mice, and 0.0313 ± 0.0074 g in the HFD-fed BL6J mice (p < 0.01 versus the CD-fed BL6J; not significant versus HFD-fed BL6N mice by an ANOVA with the LSD post-hoc test), indicating that the liver weight was increased to approximately the same level by feeding an HFD in both strains.

**Figure 5:**
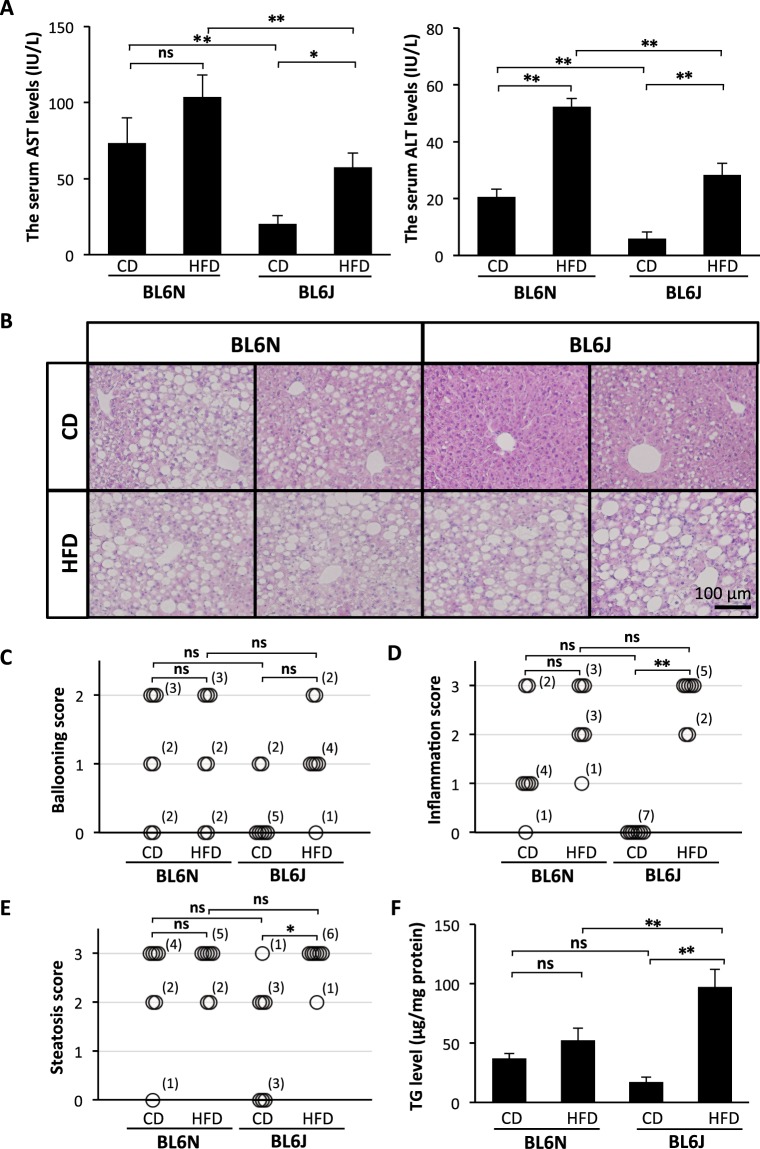
Degree of liver injury, hepatic inflammation and triglyceride accumulation in BL6J and BL6N mice on an HFD. (**A**) The serum levels of AST and ALT were determined using enzymatic assays. The bar graphs represent the means ± SE (n = 7/group). Significance was evaluated using an ANOVA with the LSD post-hoc test. *P < 0.05, **P < 0.01. (**B**) Paraffin-embedded liver sections were stained with HE. (**C–E**) Hepatocyte ballooning, hepatic inflammation and steatosis scores were evaluated as detailed in the Materials and Methods. Significance was evaluated using the Kruskal-Wallis test. *P < 0.05, **P < 0.01. (**F**) The triglyceride levels in the liver were determined using enzymatic assays. Significance was evaluated using an ANOVA with the LSD post-hoc test. *P < 0.05, **P < 0.01.

**Figure 6:**
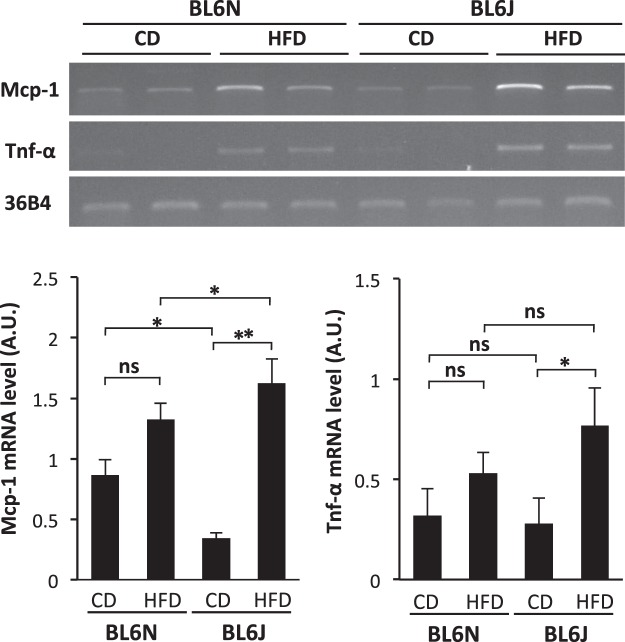
The mRNA expression of Mcp-1 and Tnf-α in BL6J and BL6N mice on an HFD. The mRNA levels of Mcp-1 and Tnf-α in the liver were determined by RT-PCR. The cropped gels were displayed in Fig. 6, and the full-length gels were presented in Supplementary Fig. 5. The band intensity was measured using the NIH ImageJ software program and normalized to that of 36B4. The data are presented as the means ± SE (n = 7/group). Significance was evaluated using an ANOVA with the LSD post-hoc test. *P < 0.05, **P < 0.01.

To assess the degree of HFD-induced liver injury in the BL6J and BL6N mice, the serum levels of AST and ALT were measured. As shown in Fig. 5A, the levels of AST and/or ALT were significantly increased by feeding an HFD in both strains, and the levels of AST and ALT in the BL6J mice were significantly lower than those of the BL6N mice. HE staining of liver sections showed that more severe steatosis was observed in the BL6J and BL6N mice fed an HFD than in the control groups; notably, even the BL6N mice consuming a CD showed fat droplets in the liver (Fig. 5B). There were no marked differences in the severity of the hepatocyte ballooning, leukocyte infiltration, or steatosis between the HFD-fed BL6J and BL6N mice (Fig. 5C–E), but the BL6J mice exhibited more distinct changes in the hepatic pathology between the CD and HFD-fed groups than did the BL6N mice. Furthermore, each mouse was categorized as “non-NASH”, “borderline NASH”, or “NASH”, based on a NAFLD activity score (NAS) system (see Methods)^1^: the respective numbers of mice with “non-NASH”, “borderline NASH” and “NASH” were 0, 2 and 5 in the CD-fed BL6N mice; 0, 1 and 6 in the HFD-fed BL6N mice; 5, 2 and 0 in the CD-fed BL6J mice; and 0, 0 and 7 in the HFD-fed BL6J mice. In addition, the hepatic triglyceride (TG) level was dramatically increased by feeding an HFD in the BL6J mice, but not in the BL6N mice (Fig. 5F). RT-PCR showed that the mRNA expression of Mcp-1, which stimulates the migration of monocytes into inflammatory sites^16–18^, was markedly increased in the liver of the BL6J mice by feeding an HFD; in contrast, the BL6N mice exhibited no marked increase in the Mcp-1 expression (Fig. 6 and Supplementary Fig. 5), likely because of the significantly higher Mcp-1 expression in the CD-fed BL6N mice than in the CD-fed BL6J mice. In addition, the Mcp-1 mRNA expression in the HFD-fed BL6J mice was significantly higher than that in the HFD-fed BL6N mice. The mRNA expression of Tnf-α, which is associated with lipid metabolism, hepatic inflammation and fibrosis in the progression of NAFLD/NASH^19,20^, was significantly increased in the BL6J mice by feeding an HFD, but there was little change in the Tnf-α expression in the BL6N mice (Fig. 6 and Supplementary Fig. 5). The extent of inflammation tended to be higher in the BL6J mice than in the BL6N mice on an HFD.

### No difference in HFD-induced hepatic fibrosis between the BL6 substrains

**Figure 7:**
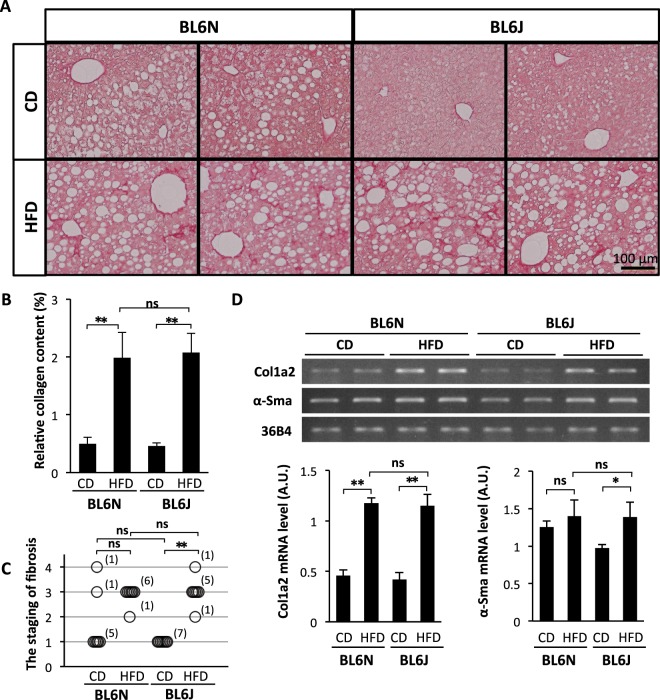
HFD-induced hepatic fibrosis in BL6J and BL6N mice. (**A**) Liver sections were stained with picrosirius red. (**B**) The relative collagen content was analyzed using the NIH ImageJ software program. (**C**) The staging of hepatic fibrosis was classified into stages 0–4. (**D**) The mRNA expression of Col1a2 and α-Sma in the liver was determined by RT-PCR. The cropped gels were displayed in (**C**), and the full-length gels were presented in Supplementary Fig. 6. The band intensity was measured using the NIH ImageJ software program and normalized to that of 36B4. The bar graphs represent the means ± SE (arbitrary units: A.U., n = 7/group). Significance was evaluated using an ANOVA with the LSD post-hoc test. ns: non-significant, *P < 0.05, **P < 0.01.

Marked collagen deposition in the liver was observed surrounding the central and portal veins in the HFD-fed BL6J and BL6N mice (Fig. 7A,B). There was, however, no marked difference in the degree and staging of hepatic fibrosis between the substrains fed an HFD (Fig. 7A–C). In addition, RT-PCR indicated that the mRNA levels of Col1a2 and α-Sma on an HFD were approximately the same in both substrains (Fig. 7D and Supplementary Fig. 6), confirming no marked difference in the progression of the hepatic fibrosis between the BL6J and BL6N mice in the HFD-induced NASH model.

### Marked liver pathology in BL6N mice on modified HFD

**Figure 8:**
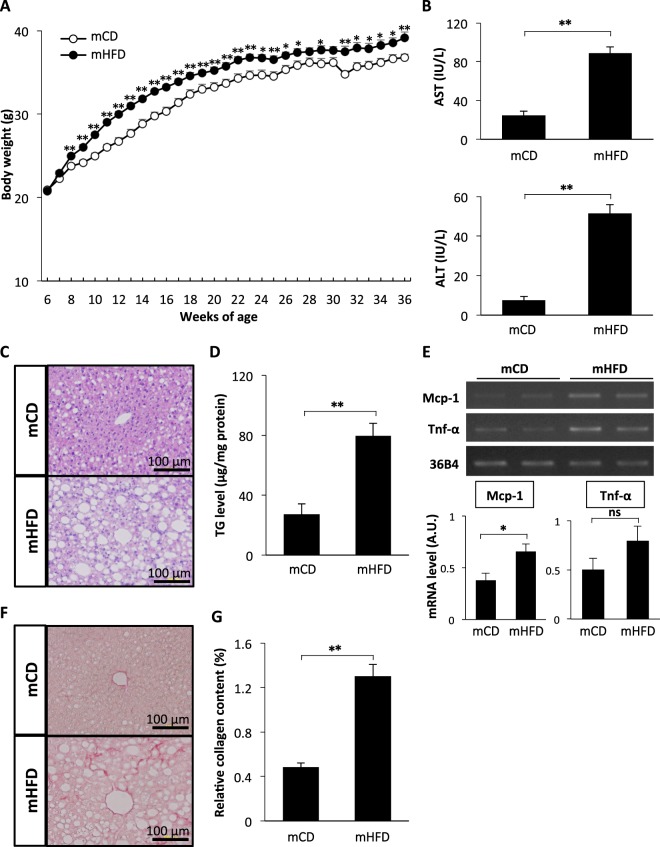
Distinctly induced hepatic pathology in BL6N mice on an mHFD. (**A**) Mice were fed a modified-control diet (mCD) or a modified high-fat diet (mHFD) for 30 weeks, and the body weights were measured every week. The data represent the means ± SE (n = 8/group). (**B**) The serum levels of AST and ALT were determined using enzymatic assays. (**C**) Liver sections were stained with HE. (**D**) The hepatic triglyceride levels were determined using enzymatic assays. (**E**) The mRNA levels of Mcp-1 and Tnf-α in the liver were determined by RT-PCR. The cropped gels were displayed in (**E**), and the full-length gels were presented in Supplementary Fig. 7. The band intensity was measured using the NIH ImageJ software program, and normalized to that of 36B4. (**F**) Liver sections were stained with picrosirius red. (**G**) The relative collagen content was analyzed using the NIH ImageJ software program. The bar graphs represent the means ± SE (arbitrary units: A.U., n = 8/group). Significance was evaluated using Student’s *t*-test. *P < 0.05, **P < 0.01.

As shown Figs 5 and 6, the BL6N mice did not exhibit the distinct changes of liver injury, hepatic inflammation, or lipid accumulation between the CD (D09100304) and HFD (D09100301)-fed groups. To determine a more appropriate diets for the BL6N mice, we next fed modified diets, an mCD (D16070901) and mHFD (D16010101), to the BL6N mice for 30 weeks (Supplementary Table 1), and analyzed the body weight and hepatic pathology. There was no marked difference in the amount of food consumed between the mCD- and mHFD-fed mice; 2.63 ± 0.05 g in the mCD-fed mice, and 2.87 ± 0.22 g in the mHFD-fed mice. A more rapid increase in the body weight was observed in the mHFD-fed mice than in the mCD-fed mice, especially over the first phase (Fig. 8A). The left lateral liver lobe weight/body weight ratios were 0.0151 ± 0.0004 g in the mCD-fed mice and 0.0378 ± 0.0012 g in the mHFD-fed mice (p < 0.01 by Student’s t-test). The serum levels of both AST and ALT were significantly higher in the mHFD-fed mice than in the mCD-fed mice (Fig. 8B). The HE-stained sections showed that the mHFD-fed mice exhibited marked steatosis and leukocyte infiltration in the liver, while the mCD-fed mice showed these effects much less markedly (Fig. 8C). In addition, the hepatic TG level was markedly increased by feeding the mHFD (Fig. 8D). The mRNA level of Mcp-1 was significantly increased by feeding an HFD, although the Tnf-α expression only showed an increasing trend (Fig. 8E and Supplementary Fig. 7). Furthermore, marked collagen deposition was observed in the liver of the mHFD-fed mice but not in that of the mCD-fed mice (Fig. 8F,G). Likewise, the mice fed the mHFD for 24 weeks exhibited higher serum levels of the serum AST and ALT, greater steatosis and TG accumulation, higher levels of Mcp-1 and Tnf-α mRNA, and more severe fibrosis in the liver than the mCD-fed mice (Supplementary Figs 8 and 9). These data suggest that, for the BL6N substrain, the mHFD and mCD are more suitable for creating an HFD-induced NASH model and the respective control than an HFD or CD.

## Discussion

According to the descriptions from breeders, the BL6J mice were introduced from The Jackson Laboratory to Charles River. In contrast, the BL6N mice (C57BL/6NCrSlc) were introduced as C57BL/6Cr to the Institute of Medical Science of The University of Tokyo, by Mr. Samuel M Poiley (National Cancer Institute of the NIH) and then transferred to Japan SLC^11^. A spontaneous loss-of-function by Nnt mutation in BL6J substrains is the most widely known genetic difference from BL6N substrains^8,9^, and 30 SNPs were found to differ between the BL6J and BL6N (C57BL/6NCrSlc) mice^11^. Our present study clearly showed the presence of phenotypic differences in the hepatology between the BL6J and BL6N (C57BL/6NCrSlc) substrains in NASH models. In the CCl_4_-induced NASH model, the BL6J mice exhibited more severe oxidative stress and fibrosis in the liver than the BL6N mice. Hepatocytes mainly expressing CYP2E1 are directly damaged and release diffusible mediators, including reactive oxygen species (ROS), which can activate HSC^21^. NNT catalyzes the reversible transfer of hydrogen between NAD and NADP and is also important in the regulation of redox reactions in the mitochondria, providing NADPH for the regeneration of antioxidants, glutathione, and thioredoxin^22–26^. Thus, the imbalance in the redox state caused by the loss-of-function of NNT protein in the BL6J mice may lead to more severe oxidative stress and subsequent fibrosis than in the BL6N mice in the CCl_4_-induced NASH model. We also showed that the hepatic inflammation score tended to be higher in the BL6J mice than in the BL6N mice on an HFD, although the degree of the hepatotoxicity was comparable between the two substrains. The higher degree of inflammation might be related to the more severe fibrosis in the BL6J mice.

Our study also demonstrated the hepatological differences between the BL6J and BL6N mice in the HFD-induced NASH model, including less severe liver injury and greater accumulation of hepatic TG in the BL6J mice than in the BL6N mice, although there was no marked difference in the degree or staging of hepatic fibrosis between the BL6 substrains. The BL6J substrain exhibits a defective insulin secretory response to intravenous glucose compared to the BL6NCrl substrain from Charles River^27,28^ and the BL6NJ substrain (C57BL/6NJ) from The Jackson Laboratory^29^ and is also characterized by a lower fat mass than the BL6NJ substrain on an HFD^29^. These findings might explain the higher levels of hepatic TG on an HFD in the BL6J mice than in the BL6N mice in the present study. In addition, because the transgenic expression of the *Nnt* gene in the BL6J mice rescues their impaired insulin secretion and glucose intolerant phonotype^30^, and the deletion of *Nnt* gene in the C57BL/6JUnib substrain aggravates HFD-induced steatosis and TG accumulation in the liver^31^, the Nnt mutation most likely influenced the increased accumulation of hepatic TG noted in the BL6J mice on an HFD in the present study. We also found that even the BL6N mice consuming a CD were susceptible to NASH, but not the BL6J mice on a CD, while the HFD-fed BL6J mice exhibited a dramatic progression of NASH compared with the BL6N mice consuming an HFD. Fisher-Wellman *et al*. showed that, under low-fat diet conditions, the insulin level during the intraperitoneal glucose tolerance test and insulin-stimulated Akt signaling activity in the liver were high in the BL6NJ substrain relative to the BL6J substrain^29^. These findings may explain the mechanism underlying the higher levels of hepatic TG in even the BL6N mice consuming a CD in the present study. As the International Knockout Mouse Consortium (IKMC) selected C57BL6N embryonic stem (ES) cells for the generation of its targeted constructs^32^, we modified the diets for the BL6N mice with the cooperation of Research Diets Inc. and found that the mHFD and mCD were particularly useful for establishing an HFD-induced NASH model and its control. Because the abnormal histology was improved by the replacement of sucrose with corn starch in the control diet (Supplementary Table 1), and given that fructose appears to play a major role in not only the initiation of hepatic steatosis but also the progression to NASH^33,34^, the BL6N substrain might be more susceptible to the progression of sucrose-induced NAFLD than the BL6J substrain.

We also compared the levels of the 13-HPODE-modified protein in the liver between the BL6J and BL6N mice on a CD and HFD; our results showed that the levels of HEL-protein adduct were not significantly different among the groups (Supplementary Fig. 10). A recent study reported that the ratio of reduced glutathione to oxidized glutathione was significantly decreased in the liver of the BL6NJ substrain on an HFD; but this effect was absent in the BL6J substrain, likely reflecting compensatory increases in alterative redox buffering pathways^29^. In contrast, the interaction between an HFD and the Nnt mutation results in redox imbalance and increased susceptibility to permeability transition pore opening in hepatic mitochondria, leading to fatty liver^31^. Thus, the Nnt mutation contributes, at least in part, to HFD-induced redox imbalance in the liver, although further research on the role of NNT in HFD-induced oxidative damage will be required to confirm these points.

Our study furthermore showed that the BL6J mice exhibited less weight gain than the BL6N mice on an HFD. Several previous studies have also detected the differences in weight regulation among substrains; for example, the BL6J substrain exhibits a more delayed increase in body weight than the C57BL/6NCrl substrain^28^, and a lower body weight was observed in the BL6J substrain than in the C57BL/6Ntac substrain from Taconic Farms on an HFD^35^. Furthermore, a normal diet-fed BL6J substrain showed less weight gain than the BL/6NJ substrain^36^. The BL6J substrain has lower level of basal serum insulin than the C57BL/6Ntac or C57BL/6NJ substrain^35,36^, and exhibits impaired glucose tolerance compared to the BL6NCrl strain^27,28^, likely explaining a cause of less weight gain in the BL6J substrain than in the BL6N substrains. However, the difference in weight regulation and insulin secretion cannot be explained by the Nnt mutation alone^36–39^; therefore, the relationship between the genetic variants and the phenotypic differences among substrains needs to be studied further.

This study elucidated for the first time the pathological differences between the BL6J and BL6N substrains in the CCl_4_- and HFD-induced NASH models. As genetic drift occurs within an isolated breeding population and the phenotypic effects of the genetic variants among C57BL/6 substrains are still unclear, the appropriate use of substrains from the same vendor as controls are recommended for studies related to the progression of NAFLD/NASH. The Cre recombinase/loxP system has been extensively used in recent years to generate conditional (i.e. tissue- or cell-type–specific) genetically modified mice, in which heterozygous or homozygous floxed mice are bred with Cre-recombinase transgenic mice^40,41^. As most of the floxed mice for various genes and the available Cre-recombinase transgenic mice have been generated using targeted ES cells from the BL6J and BL6N substrains^9,42,43^, the resultant conditional genetically modified mice can have a mixed background. The findings from the current study strongly suggest that the genetic background be taken into careful consideration, especially when generating conditional knockout mice.

## Methods

### Animals

Male five-week-old C57BL/6J (BL6J) mice and C57BL/6NCrSlc (BL6N) mice were purchased from Japan Charles River (Yokohama, Japan) and from Japan SLC (Hamamatsu, Japan), respectively. According to the descriptions from Japan Charles River, BL6J mice were introduced from The Jackson Laboratory and bred in accordance with The Jackson Laboratory genetic management system. This means that the BL6J mice bred by Japan Charles River are equivalent in genetic quality to those bred by The Jackson Laboratory. Mekada *et al*. reported that 30 SNPs differ between BL6J mice from The Jackson Laboratory and BL6N mice from Japan SLC^11^ and that the deletion of exons 7–11 in the *Nnt* gene is detected in BL6J mice but not in BL6N mice^44^. We confirmed the absence of Nnt mRNA expression in C57BL/6J but not in C57BL/6NCrSlc (Supplementary Fig. 11). All mice were housed at <5/cage with a 12-h light-dark cycle and *ad libitum* access to food and water. All experiments were approved by the institutional animal care and use committee of Kyoto Pharmaceutical University (Permit number: 18-13-036), and were performed in accordance with the institutional guidelines.

### Animal models

For a model of CCl_4_-induced hepatic fibrosis, six-week-old BL6J and BL6N mice were randomly divided into two groups: a control group and a CCl_4_ administered group. Liver fibrosis was induced by twice weekly intraperitoneal administration of CCl_4_ at 0.31 mL/kg body weight (diluted in corn oil; Sigma-Aldrich, St. Louis, MO, USA) for 6 weeks. For a model of high-fat diet-induced NASH, 6-week-old BL6J and BL6N mice were randomly divided into a control-diet (CD) group and a high-fat-diet (HFD) group and fed either a control diet (4.3% fat; D09100304; Research Diets Inc., NJ, USA) or a high-fat diet (19.9% fat and 2.0% cholesterol; D09100301; Research Diets Inc.), respectively, for 30 weeks. To make the hepatological changes in the BL6N mice more distinct, the sucrose in the CD was replaced with corn starch, resulting in modified-CD (mCD; 4.3% fat; D16070901; Research Diets Inc.). In addition, the partially hydrogenated soybean and palm oils in HFD were replaced with partially hydrogenated corn oil, resulting in modified-HFD (mHFD; 20% fat and 2.0% cholesterol; D16010101; Research Diets Inc.) (Supplementary Table 1). The 6-week-old BL6N mice were randomly divided into an mCD group and an mHFD group and fed either mCD or mHFD for 24 and 30 weeks.

### Serum biomarker measurements

Blood samples were collected from the inferior vena cava and allowed to stand for 1 h, and then the serum was prepared by centrifugation at 10,000 × g for 10 min at room temperature. The activities of serum alanine aminotransferase (ALT) and aspartate aminotransferase (AST) were measured using Transaminase C II-Test kits (Wako Pure Chemical Industries, Osaka, Japan).

### Histological analyses

The mice were transcardially perfused with saline and 10% buffered formaldehyde, and the livers were then post-fixed in 10% buffered formaldehyde for 48 h. The fixed livers were embedded in paraffin for microtome slicing into 5-μm-thick sections. The tissue sections were mounted onto MS-coated glass slides, deparaffinized, and stained with picric acid-Sirius red or hematoxylin and eosin (HE; Wako Pure Chemical Industries). Stained sections were photographed using a microscope (model IX71; Olympus, Tokyo, Japan) with a digital camera. Images were taken at full resolution with a single image dimension set at 1,360 × 1,024 pixels. HE-stained specimens were scored for the severity of hepatocellular ballooning, inflammation, and steatosis according to the following criteria: for inflammatory cell infiltration, specimens were classified into grades 0–3 (grade 0: none; grade 1: 1 focus per 200x field; grade 2: 2–4 foci per 200x field; and grade 3: ≥5 foci per 200x field); for hepatocellular ballooning, specimens were classified into grades 0–2 (grade 0: none; grade 1: 1–4 balloon cells; and grade 2: ≥5 prominent ballooning cells); for hepatocellular steatosis, specimens were classified into grades 0–3 (grade 0: <5%; grade 1: steatosis occupying 5–33% of the hepatic parenchyma; grade 2: 33–66% of the hepatic parenchyma; and grade 3: ≥66% of the hepatic parenchyma)^1^. The NAFLD activity score (NAS)—the sum of hepatocellular steatosis, inflammation and ballooning scores—was used to diagnose “NASH”, “borderline NASH” or “non-NASH” as follows: a NAS of ≥5 was defined as “NASH”, a NAS of 3 or 4 was defined as “borderline NASH”, and a NAS of <3 was defined as “non-NASH”. The staging of hepatic fibrosis was classified into stages 0–4 (stage 0: none; stage 1: mild or moderate, perisinusoidal or periportal fibrosis; stage 2: perisinusoidal and periportal fibrosis; stage 3: bridging fibrosis; and stage 4: cirrhosis)^1^. Collagen fibers stained with Sirius red were quantified by measuring the red areas using the U.S. National Institutes of Health ImageJ v1.47 software program (http://rsb.info.nih.gov/ij).

### Reverse transcription-polymerase chain reaction (RT-PCR)

Total RNA was extracted from the liver using an RNAiso Plus (Takara Bio, Shiga, Japan) according to the manufacturer’s instructions. Total RNA (5 μg) was reverse-transcribed using dNTPs, random primers and ReverTra Ace reverse transcriptase (Toyobo, Osaka, Japan). PCR was then performed on cDNA samples using Blend Taq DNA polymerase or KOD FX DNA polymerase (Toyobo, Osaka, Japan). We used the following primer sets: Cyp2e1, 5′-AGTGTTCACACTGCACCTGG-3′ (sense) and 5′-CCTGGAACACAGGAATGTCC-3′ (antisense); Col1a2, 5′-CCGTGCTTCTCAGAACATCA-3′ (sense) and 5′-CTTGCCCCATTCATTTGTCT-3′ (antisense); smooth muscle α-actin (α-Sma), 5′-CAGCGGGCATCCACGAA-3′ (sense) and 5′-GCCACCGATCCAGACAGA-3′ (antisense); Mcp-1, 5′-AGGTCCCTGTCATGCTTCTG-3′ (sense) and 5′-TCTGGACCCATTCCTTCTTG-3′ (antisense); Tnf-α, 5′-GGCAGGTCTACTTTGGAGTCATTGC-3′ (sense) and 5′-ACATTCGAGGCTCCAGTGAATTCGG-3′ (antisense); 36B4, 5′-TGTGTGTCTGCAGATCGGGT-3′ (sense), 5′-TGGATCAGCCAGGAAGGCCT-3′ (antisense). The PCR products were analyzed on a 1.5% agarose gel and visualized by ethidium bromide staining. The band intensities were quantified using the ImageJ software program. The expression was normalized to that of 36B4 RNA.

### Immunoblotting

Liver specimens were homogenized in ice-cold lysis buffer containing 50 mM Tris–HCl (pH 7.4), 150 mM NaCl, 1% Nonidet P-40, 1% sodium deoxycholate, and 0.1% sodium dodecyl sulfate supplemented with a complete protease inhibitor cocktail tablet (Roche Diagnostics, Indianapolis, IN, USA) and phosphatase inhibitor cocktail solution (Wako Pure Chemical Industries). Protein extracts were collected after centrifugation at 10,000 × g for 20 min at 4 °C. The protein concentrations were determined using the Bradford method. The lysates containing equal amounts of protein were denatured and subjected to SDS–polyacrylamide gel electrophoresis on a 10% acrylamide gel. Proteins were transferred onto nitrocellulose membranes. After being blocked with 3% skim milk in Tris-buffered saline containing 0.05% Tween-20 (TBS-T), the membranes were incubated with antibodies against hexanoyl-lysine (HEL) (Japan Institute for the Control of Ageing, Shizuoka, Japan; diluted 1:500 with blocking solution), α-SMA (Sigma-Aldrich; diluted 1:1000 with blocking solution), or GAPDH (Wako Pure Chemical Industries; diluted 1:4000 with blocking solution) at 4 °C overnight. After being washed with TBS-T, the membranes were incubated with horseradish peroxidase-conjugated goat anti-mouse IgG (Santa Cruz Biotechnology; diluted 1:2500 with TBS-T or 0.3% skim milk in TBS-T) for 1 h. After being washed again, immunoreactive bands were detected using Chemi-Lumi One Super (Nacalai Tesque, Kyoto, Japan) with an LAS-3000 mini-image analysis system (Fujifilm, Tokyo, Japan). The band intensities were quantified using the ImageJ software program.

### Measurement of hepatic lipid contents

Liver specimens (50 mg) were homogenized in H_2_O containing protease inhibitors, and hepatic lipids were extracted using chloroform-methanol (1:1, v/v) and 0.1 M KCl. The lipid extracts were mixed with 2-methyl-2-propanol and Triton-100-methanol (1:1, v/v), and the hepatic triglyceride level was then determined using Triglyceride E-test Wako kits (Wako Pure Chemical Industries).

### Statistical analyses

Data are indicated as the mean ± standard error of means (SE). Differences among means were analyzed using a one-way analysis of variance (ANOVA) followed by an LSD post-hoc test, Kruskal-Wallis test, or Student’s *t*-test. P < 0.05 was considered as the lowest level of significance.

## Supplementary information

Supplementary information
