# Supplementary material for: A comparative analysis of hepatic pathological phenotypes in C57BL/6J and C57BL/6N mouse strains in non-alcoholic steatohepatitis models
Source: Sci Rep. 2019 Jan 18;9:204. doi: 10.1038/s41598-018-36862-7 (PMC6338790; doi:10.1038/s41598-018-36862-7)
Supplement: Supplementary file 1 — Supplementary information [file 41598_2018_36862_MOESM1_ESM.pdf]

Supplementary information

A comparative analysis of hepatic pathological phenotypes in C57BL/6J and C57BL/6N mouse strains in non-alcoholic steatohepatitis models

Eri Kawashita*, Keiichi Ishihara*, Madoka Nomoto*, Mika Taniguchi*, Satoshi Akiba*

*Department of Pathological Biochemistry, Kyoto Pharmaceutical University,

5 Misasaginakauchi-cho, Yamashina-ku, Kyoto 607-8414, Japan.

Supplementary Figure 1. The mRNA levels of Cyp2e1 in a CCl_4-induced NASH model

The mRNA levels of Cyp2e1 in the livers of BL6J and BL6N mice were determined by RT-PCR. The band intensity was measured using NIH ImageJ software program, and normalized to that of 36B4. The bar graphs represent the means ± SE (arbitrary units: A.U., n = 8–10/group). Significance was evaluated using an ANOVA with the LSD post-hoc test. *P < 0.05, **P < 0.01, ns: non-significant.

Supplementary Figure 2. Oxidative stress in a CCl_4-induced NASH model

The protein levels of 13-HPODE-adducted protein and GAPDH in the livers of BL6J and BL6N mice were determined by Western blotting. The full-length blots were presented.

Supplementary Figure 3. The mRNA levels of Col1a2 and α-Sma in a CCl_4-induced NASH model

The mRNA levels of Col1a2, α-Sma and 36B4 in the livers of BL6J and BL6N mice were determined by RT-PCR. The full-length gels were presented.

Supplementary Figure 4. The protein expression of α-SMA in a CCl_4-induced NASH model

The protein levels of α-SMA and GAPDH in the livers of BL6J and BL6N mice were determined by Western blotting. The full-length blots were presented.

Supplementary Figure 5. The mRNA levels of Mcp-1 and Tnf-α in an HFD-induced NASH model

The mRNA levels of Mcp-1, Tnf-α and 36B4 in the livers of BL6J and BL6N mice were determined by RT-PCR. The full-length gels were presented.

Supplementary Figure 6. The mRNA levels of Col1a2 and α-Sma in an HFD-induced NASH model

The mRNA levels of Col1a2, α-Sma and 36B4 in the livers of BL6J and BL6N mice were determined by RT-PCR. The full-length gels were presented.

Supplementary Figure 7. The mRNA levels of Mcp-1 and Tnf-α in BL6N mice fed an mHFD for 30 weeks

The mRNA levels of Mcp-1, Tnf-α and 36B4 in the liver were determined by RT-PCR. The full-length gels were presented.

Supplementary Figure 8. Hepatic pathology in BL6N mice fed an mHFD for 24 weeks

(A) Mice were fed an mCD and mHFD for 24 weeks. The serum levels of AST and ALT were determined using enzymatic assays. The data represent the means ± SE (n = 7/group). (B) Liver sections were stained with HE. (C) The hepatic triglyceride levels were determined using enzymatic assays. (D) mRNA levels of Mcp-1 and Tnf-α in the liver were determined by RT-PCR. The cropped gels were displayed in Fig. 8D, and the full-length gels were presented in Supplementary Fig. 9. The band intensity was measured using NIH ImageJ software program, and normalized to that of 36B4. (E) Liver sections were stained with picrosirius red. (F) The relative collagen content was analyzed using the NIH ImageJ software program. The bar graphs represent the means ± SE (arbitrary units: A.U., n = 7/group). Significance was evaluated using Student's t-test. *$P < 0.05$, **$P < 0.01$.

Supplementary Figure 9. The mRNA levels of Mcp-1 and Tnf-α in BL6N mice fed an mHFD for 24 weeks

The mRNA levels of Mcp-1, Tnf-α and 36B4 in the liver were determined by RT-PCR. The full-length gels were presented.

Supplementary Figure 10. Oxidative stress in an HFD-induced NASH model

The protein levels of 13-HPODE-adducted protein and GAPDH in the liver of BL6J and BL6N mice were determined by Western blotting. The full-length blots were presented.

Supplementary Figure 11. The absence of Nnt mRNA in BL6J mice, but not in BL6N mice

The absence of Nnt mRNA in the liver was confirmed by RT-PCR using the methods of Huang et al[23]. The RT-PCR analysis was performed using sense and antisense primers which are located within the coding sequence for exons 7-11. We used the following primer set: 5'-GCAGGTCTCACTGGGA-3'

(sense) and 5’-AACCAGAGATGGCATTGG-3’ (antisense); and the primers amplified a PCR product of 168 bp from BL6N mice and no product from BL6J mice. The mRNA levels of 36B4 in the liver were also determined. The full-length gels were presented.

Supplementary Table 1. The compositions of the high-fat and control diets used in the HFD-induced NASH models

HFD, high-fat diet; CD, control diet; mHFD, modified HFD; mCD, modified CD

The compositions of CD (Research Diet’s D09100304), mCD (D16070901), HFD (D09100301) , and mHFD (D16010101) are shown. The differences in the composition between CD and mCD and between HFD and mHFD are indicated by underlining. Each diet contains 50 mg of food-grade dyes added per 4057 kcals to distinguish the diets visually from one another.

.

Supplementary Fig. 1

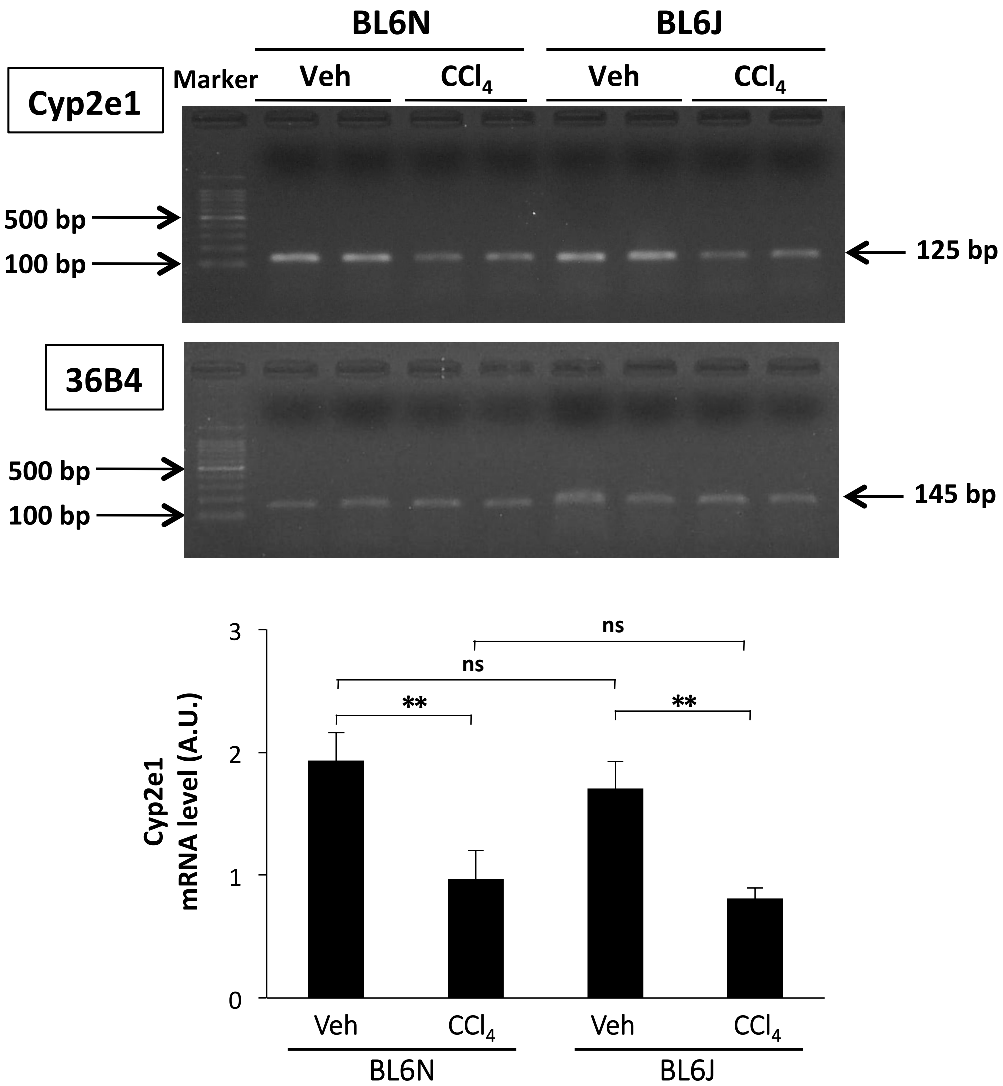

Supplementary Fig. 2

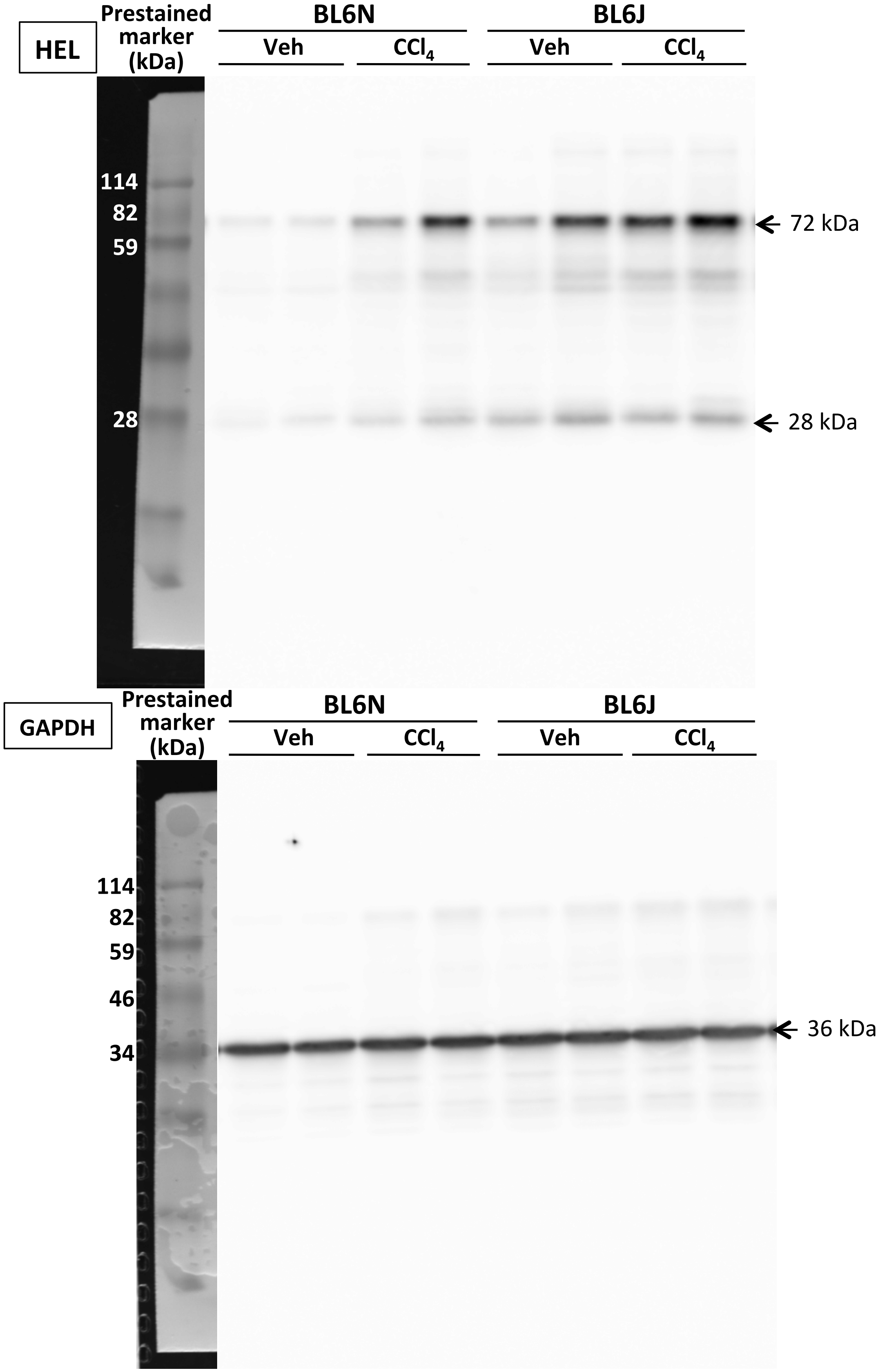

Supplementary Fig. 3

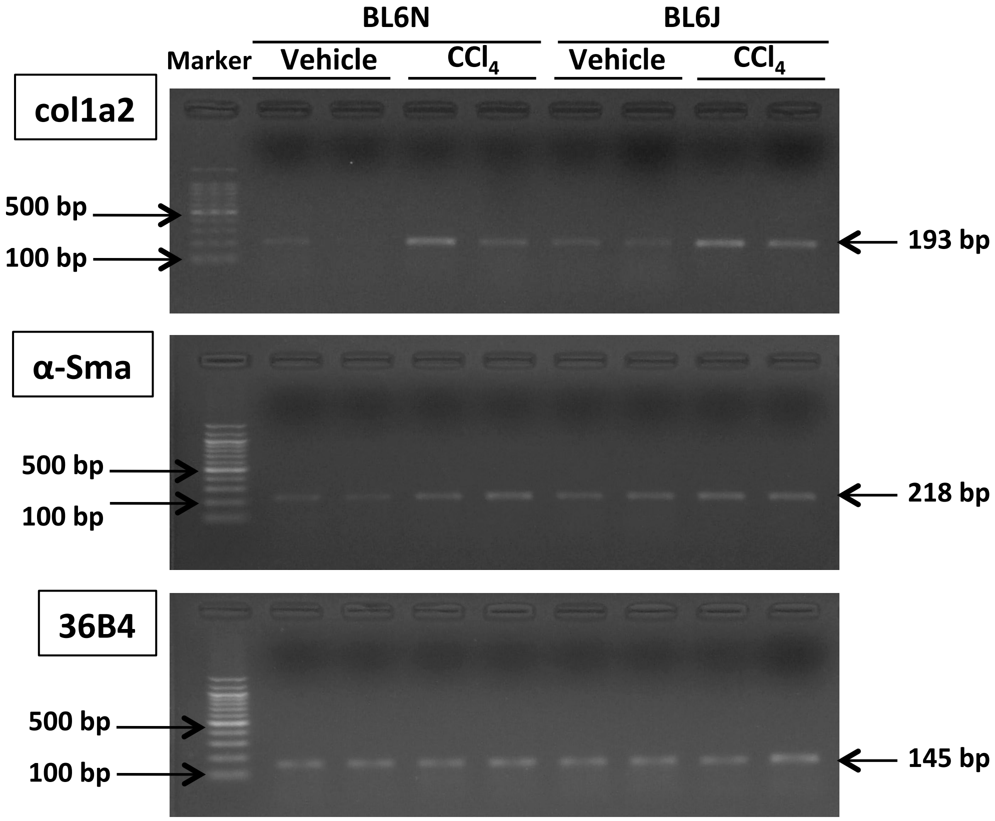

Supplementary Fig. 4

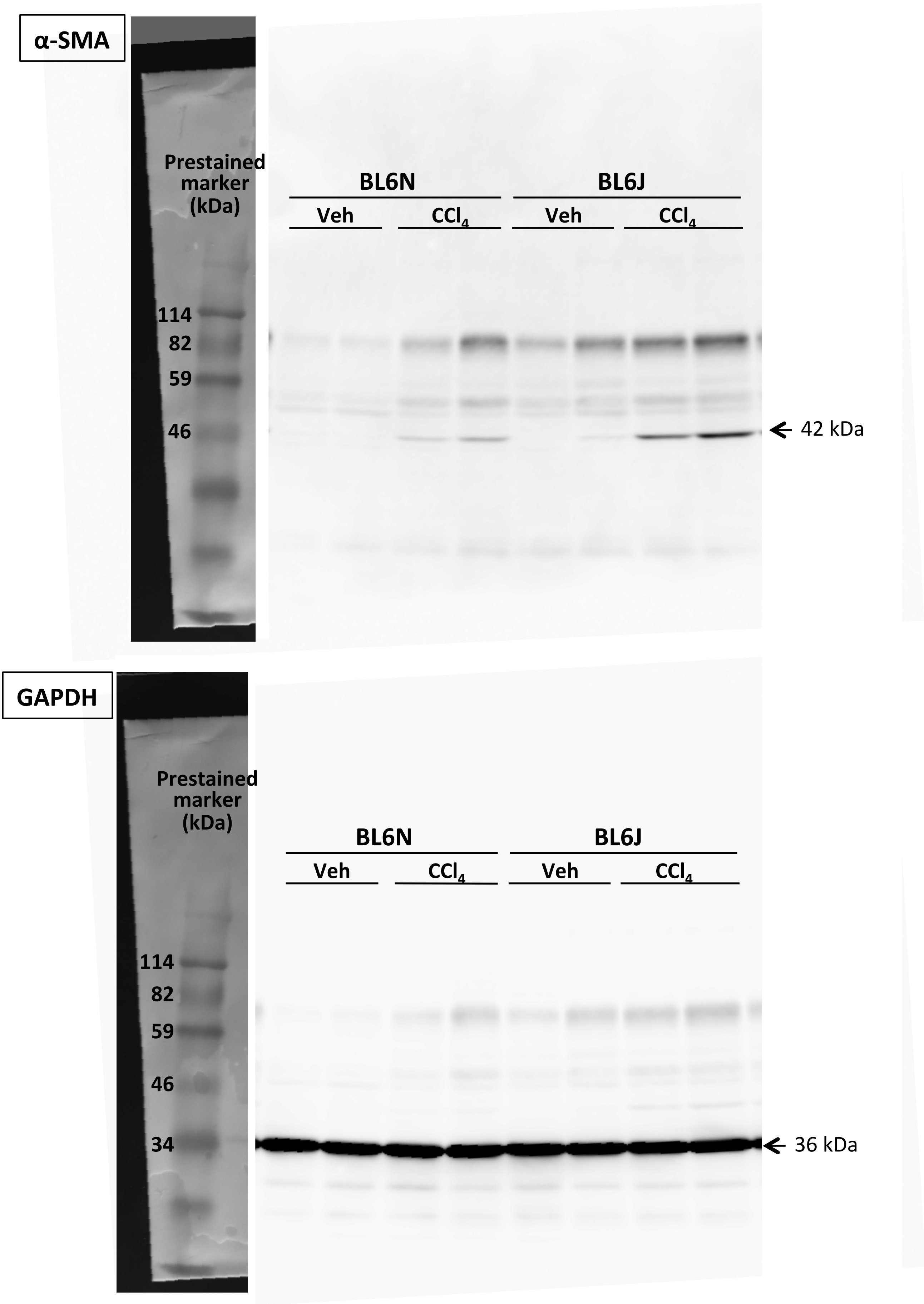

Supplementary Fig. 5

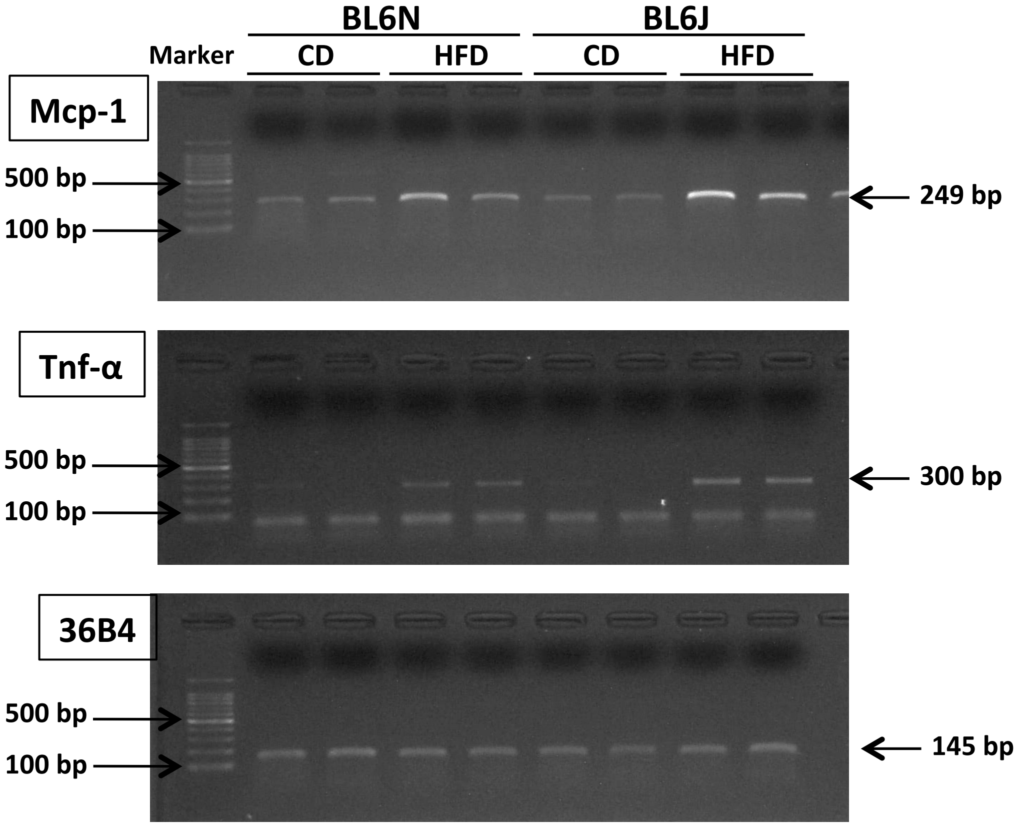

Supplementary Fig. 6

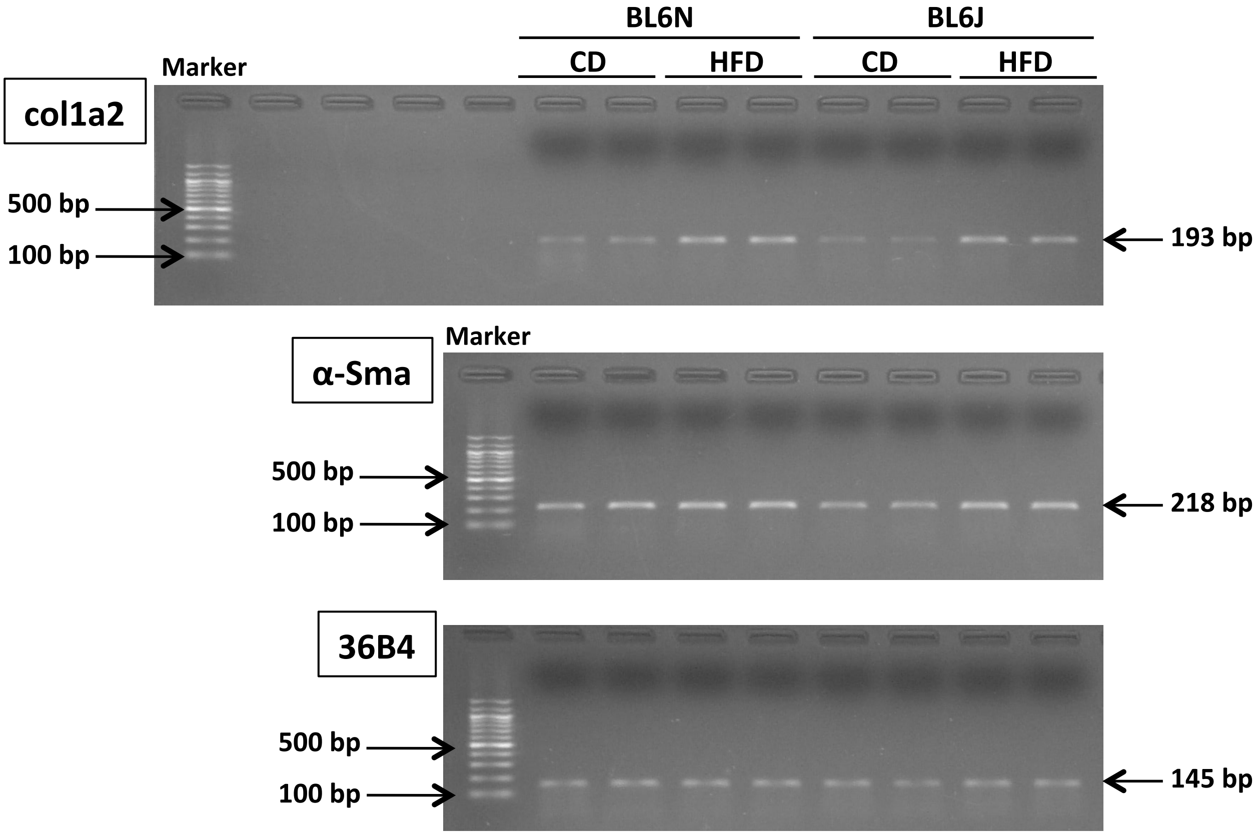

Supplementary Fig. 7

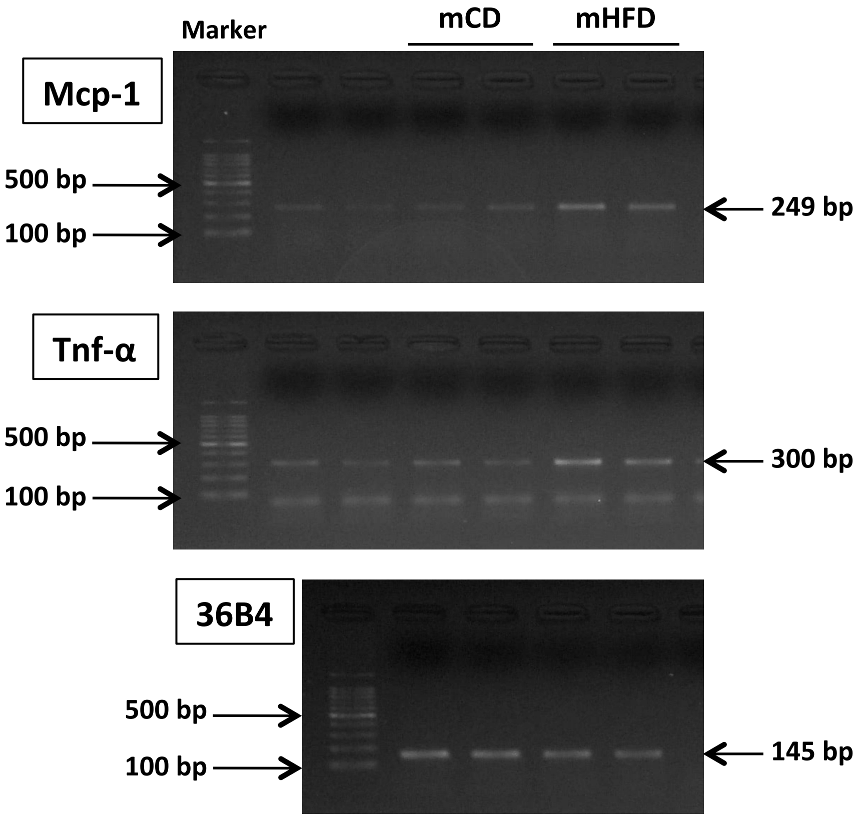

Supplementary Fig. 8

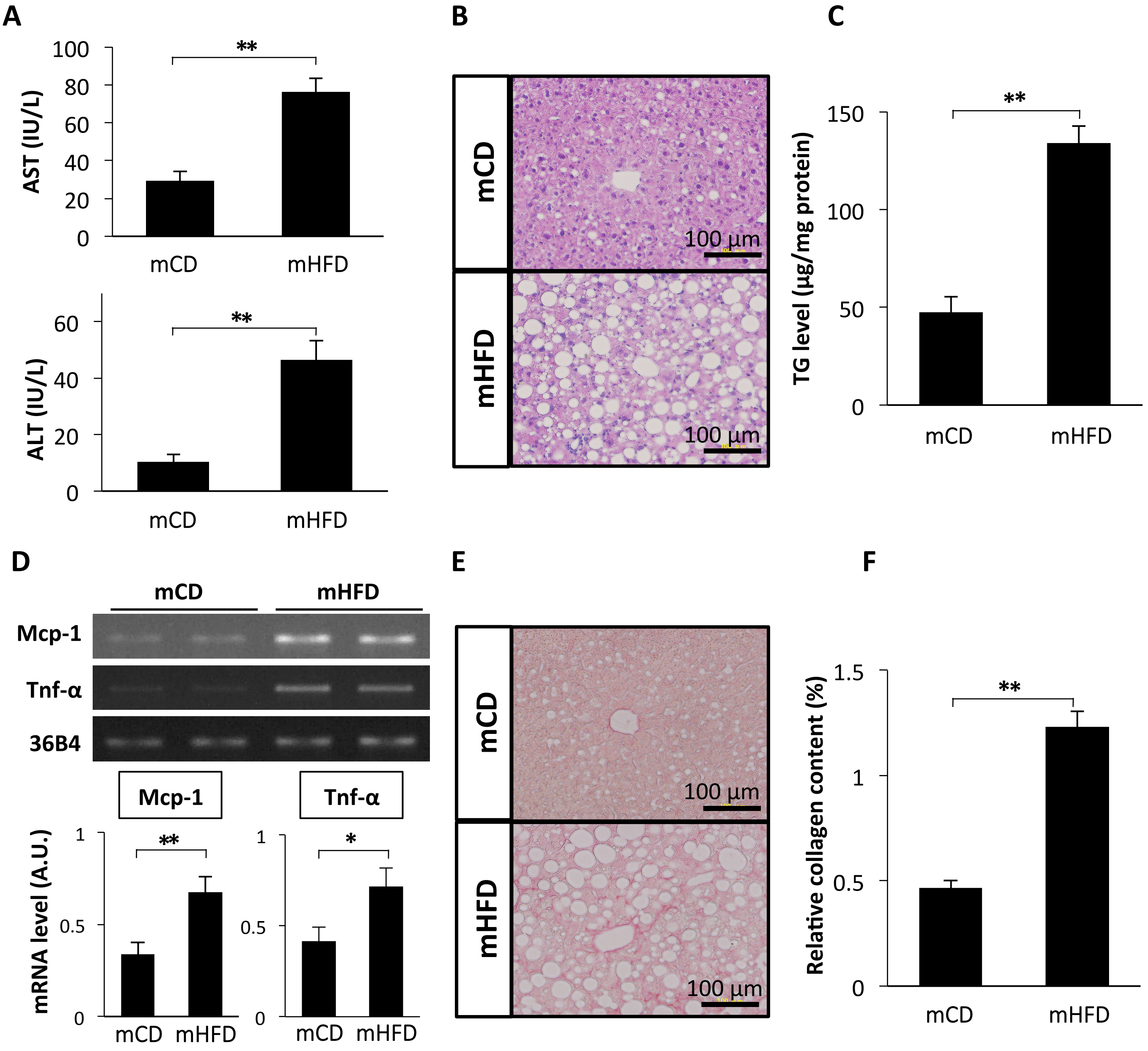

Supplementary Fig. 9

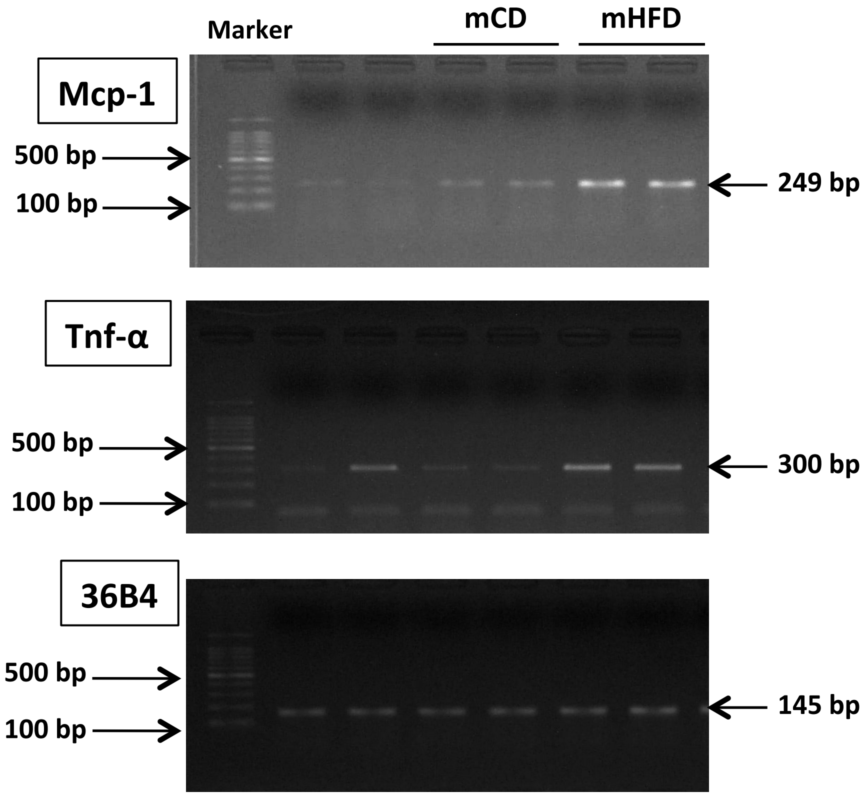

Supplementary Fig. 10

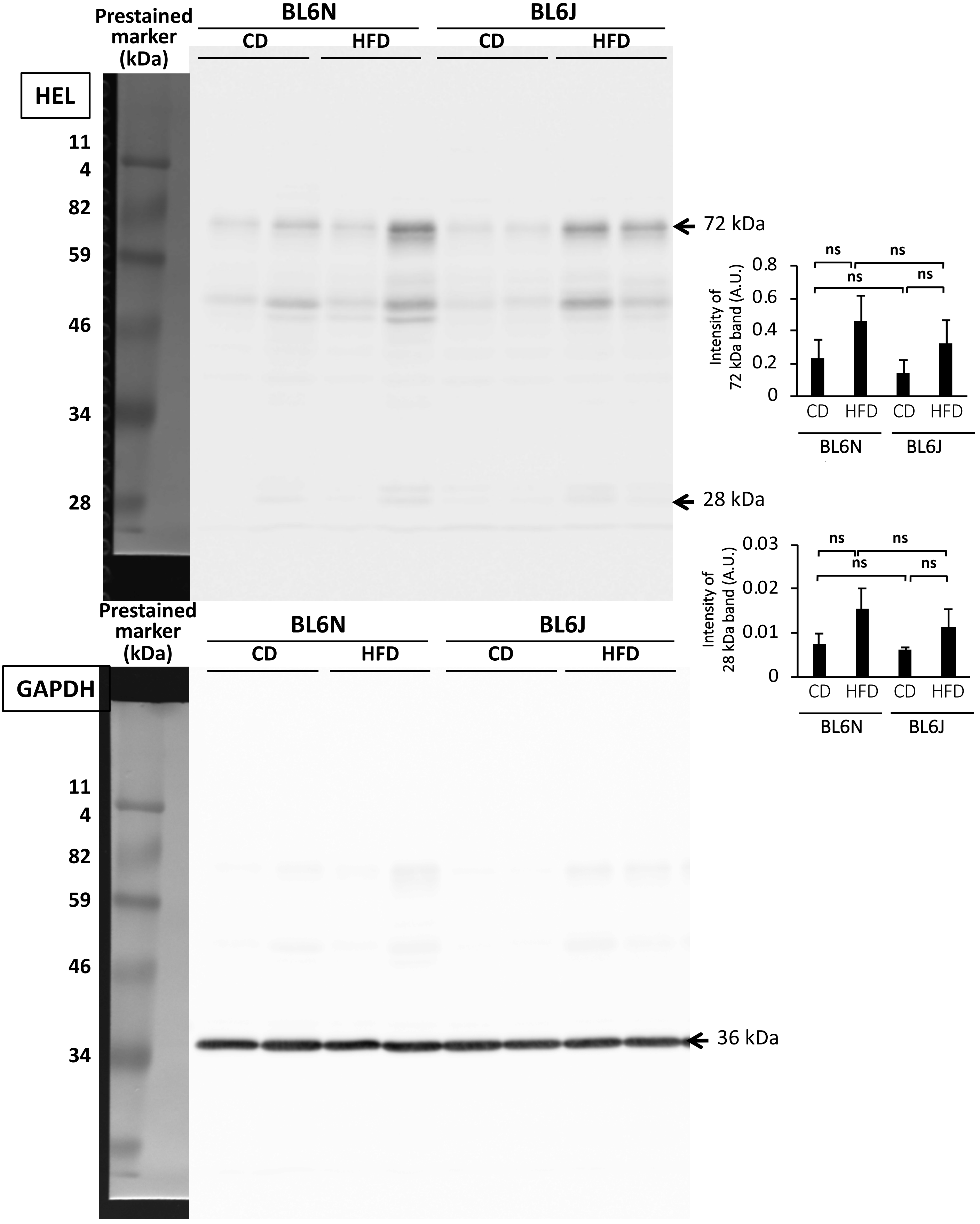

Supplementary Fig. 11

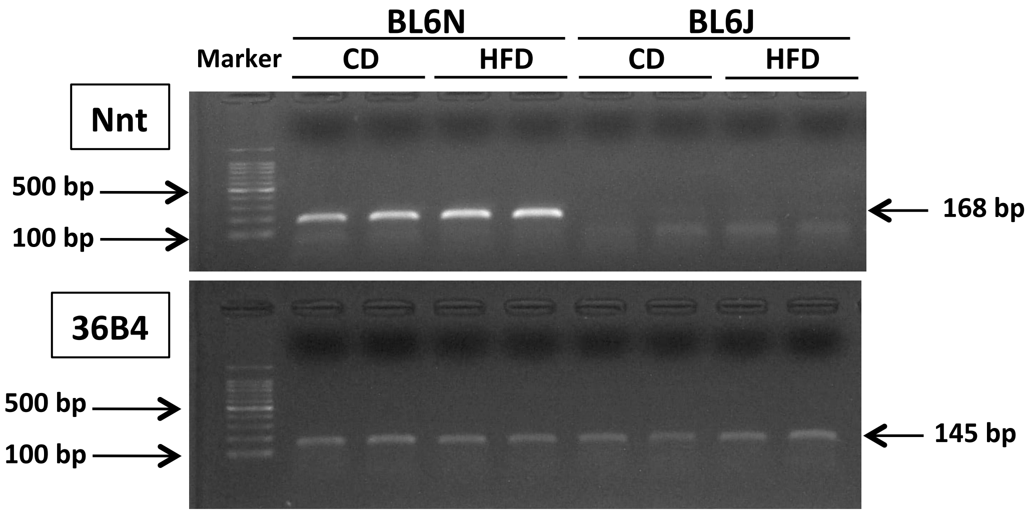

Supplementary Table 1

Diet (Product #)	CD (D09100304)		mCD (D16070901)		HFD (D0910030)		mHFD (D16010101)	
	gm%	kcal%	gm%	kcal%	gm%	kcal%	gm%	kcal%
Protein	19.2	20	19.2	20	22	20	22	20
Carbohydrate	67.3	70	67.3	70	45	40	45	40
Fat	4.3	10	4.3	10	20	40	20	40
Total		100		100		100		100
kcal/gm	3.85		3.85		4.5		4.5	
**Ingredient**	**gm**	**kcal**	**gm**	**kcal**	**gm**	**kcal**	**gm**	**kcal**
Casein	200	800	200	800	200	800	200	800
L-Cystine	3	12	3	12	3	12	3	12
Corn Starch	*350*	*1400*	*446*	*1784*	0	0	0	0
Maltodextrin 10	85	340	85	340	100	400	100	400
Glucose	169	676	169	676	0	0	0	0
Fructose	0	0	0	0	200	800	200	800
Sucrose	*96*	*384*	*0*	*0*	96	384	96	384
Cellulose	50	0	50	0	50	0	50	0
Soybean Oil	25	225	25	225	25	225	25	225
Soybean and Palm Oils, Partially Hydrogenated	0	0	0	0	*135*	*1215*	*0*	*0*
Corn Oil, Partially Hydrogenated	0	0	0	0	*0*	*0*	*135*	*1215*
Lard	20	180	20	180	20	180	20	180
Mineral Mix S10026	10	0	10	0	10	0	10	0
DiCalcium Phosphate	13	0	13	0	13	0	13	0
Calcium Carbonate	5.5	0	5.5	0	5.5	0	5.5	0
Potassium Citrate, 1 H2O	16.5	0	16.5	0	16.5	0	16.5	0
Vitamin Mix V10001	10	40	10	40	10	40	10	40
Choline Bitartrate	2	0	2	0	2	0	2	0
Cholesterol	0	0	0	0	18	0	18	0
**Total**	**1055**	**4057**	**1055**	**4057**	**904**	**4056**	**904**	**4056**
